# Beyond hallmarks of aging – biological age and emergence of aging networks

**DOI:** 10.31491/APT.2025.03.166

**Published:** 2025-03-28

**Authors:** S. Michal Jazwinski, Sangkyu Kim, Jessica Fuselier

**Affiliations:** a Tulane Center for Aging, Deming Department of Medicine, Tulane University School of Medicine, New Orleans, Louisiana 70112 USA

**Keywords:** Aging, hallmarks of aging, frailty index, biological age, aging networks, entropy

## Abstract

The hallmarks of aging have contributed immensely to the systematization of research on aging and have influenced the emergence of geroscience. The developments that led to the concepts of the hallmarks and geroscience were first marked by the proliferation of ‘theories’ of aging, mostly based on the experimental predilections of practitioners of aging research. Deeper consideration of the concepts of hallmarks of aging and geroscience leads to the quandary of whether a biological aging process exists beyond disease itself. To address this difficulty, a metric of biological age as opposed to calendar age is necessary. Several examples of biological age measured using similar assumptions, but different methods, exist. One of these, the frailty index was the first to successfully characterize aging in terms of loss of integrated function, and it is simpler than and superior to other constructs for measuring biological age. Though relatively simple in construction, the frailty index is rich conceptually, however, pointing to a network model of the aging organism. This network functions as a nonlinear complex system that is governed by stochastic thermodynamics, in which loss of integration leads to increasing entropy. Its structure transcends all levels of biological organization, such that its parts form hierarchies that are self-similar (fractal). The hallmarks of aging are simply nodes in the aging network, which can be found repetitively in various locations of the network. Stochastic thermodynamics implies that the aging system with higher entropy can exist in a multitude of possible microstates that are tantamount to high disorder with a high probability to assume a certain state. This explains the observed variability among aging individuals.

## Introduction

The past decade has brought the biology of aging to a new level of sophistication due to the emergence of geroscience and the enumeration of the hallmarks of aging. Geroscience posits that the major risk factor for chronic diseases of aging is the biological aging process [1]. The hallmarks of aging, in turn, are postulated to be the expressions of the biological aging process, at the minimum, if not the outright cause [2]. Thus, geroscience and the hallmarks of aging are intimately intertwined. This has led to the nearly religious fealty of authors to these twin concepts manifested by their ubiquitous invocation in publications in the aging research field.

The dynamic displayed by the reliance on geroscience and the hallmarks of aging to create order within the seeming randomness and disorder of aging recapitulates the earlier use of other heuristic devices. The Gompertz equation and its declaration of aging as an exponential increase in mortality was perhaps the earliest such contrivance [3]. This was given ‘flesh and bones’ by the definition of aging as the ‘progressive decline in function ability that results in decreased resilience, predisposing the organism to stress, damage, and disease, thus leading to its ultimate demise.’ At the same time, the evolutionary concept of ‘antagonistic pleiotropy’ and its physiological twin the ‘disposable soma’ stepped up to supply existential meaning to the aging process [4].

There have been many theories of aging expounded during the modern era beginning in the early 20^th^ century. Their number has been dictated by the number of researchers whose focus was a particular physiological or biochemical process that changes with age. This multiplication of age changes, rising to the conceptual level of theories, was hardly satisfying. The tipping point was reached in 1987 when the book “Modern Biological Theories of Aging” was published [5]. In this volume, several of the prevailing “theories” were expounded in a chapter by an invited proponent, and this was followed by a chapter written by an opponent. Fortunately, aging research was rescued by the vigorous emergence of the new genetics of aging which demonstrated that a change in even a single gene can extend longevity [6].

Today, we are again witnessing an increase in the explanatory factors for aging. The original nine hallmarks of aging are now twelve in number [7]. Restraint is still evident, however, because the information content of each hallmark is extensive. There is also the realization that these hallmarks are ‘connected’; they do not operate in isolation. If the past is prologue, we will see further expansion of hallmarks, nevertheless. Such a development would be unsatisfying for a science that claims some maturity by relying on a strong theoretical framework.

In this article, we discuss the mutual relationship between the hallmarks of aging and the geroscience hypothesis. This close relationship leads us to postulate that to be able to distinguish the biological aging process from age-related diseases it is necessary to be able to quantitate biological age as opposed to calendar age. We feature the deficit index, better known as the frailty index, as a useful measure of biological age, and describe its characteristics. The utility of the frailty index as a metric of biological age is highlighted in application to studies of energy metabolism, genetics, epigenetics, and metagenetics of aging. The frailty index is then juxtaposed to other methods of determining biological age, notably epigenetics clocks. The frailty index allows us to identify and to characterize the individual phenotypic variability in biological aging. The features of the frailty index lead us to postulate a network model of the complex aging system, grounded in nonlinear dynamics. In this model, biological aging is the decrease in the connectivity of the network which compromises the system’s integration and thus its coordinated functioning. The internal operations of this aging network will be best described by stochastic thermodynamics.

## Hallmarks of aging and geroscience

In their publication of the “The hallmarks of aging,” Lopez-Otin *et al.* [7] clearly define the biological aging process as a “progressive loss of physiological integrity, leading to impaired function and increased vulnerability to death”. Without naming it as such, they also enunciate the geroscience hypothesis by stating that aging is: “the primary risk factor for major human pathologies, including cancer, diabetes, cardiovascular disorders, and neuro-degenerative diseases.” The nine hallmarks are genomic instability, telomere attrition, epigenetic alterations, loss of proteostasis, deregulated nutrient sensing, mitochondrial dysfunction, cellular senescence, stem cell exhaustion, and altered intercellular communication. The authors also allude to the interconnectedness of these hallmarks.

The seven pillars of aging paper [1], published one year later, mentions geroscience explicitly, defining it in terms of the risk of chronic, age-related diseases. The list of pillars is very similar to the hallmarks of aging, encompassing adaptation to stress, epigenetics, inflammation, macromolecular damage, proteostasis, stem cells and regeneration, and metabolism. Again, interconnectedness among the pillars is emphasized. Inflammation is a pillar that isn’t featured among the hallmarks, and it constitutes a significant addition. The point of departure for this publication enumerating the seven pillars is the National Institutes of Health (NIH) mission to address the major diseases that contribute to human morbidity and mortality. This mission is combined with the goal of basic aging research to extend healthspan and with the conviction that the major risk factor for most diseases that limit healthspan is the aging process, to arrive at geroscience. Significantly, this realization led to the creation of the trans-NIH Geroscience Interest Group (GSIG), an umbrella for most of the NIH institutes and centers whose purview heretofore were aging of individual body systems and associated diseases.

Recently, the hallmarks of aging have been expanded to twelve by the addition of disabled macroautophagy, chronic inflammation, and dysbiosis [7]. It is postulated that these hallmarks of aging are interconnected with each other, and they possess connections to the hallmarks of health, which are stated to include organizational features of spatial compartmentalization, maintenance of homeostasis, and adequate responses to stress.

It will be interesting to see whether we will continue the expansionary phase in the development of the hallmarks of aging or there will be a consolidation. As will be seen, we expect that consolidation, when it happens, will be based on integrative models of the aging system that are grounded on the principles of connectivity and complexity, nonlinear dynamics and emergence, self-similarity (fractality), and bi-directional interactions with the environment.

## The biological aging process and diseases of aging, an inseparable pair

The conceptual framework provided by the hallmarks of aging is matched by the one generated by the geroscience hypothesis. Age is the major risk factor for chronic disease. Because chronic disease can accelerate aging in something akin to a feedback mechanism, it is difficult to separate the two. Indeed, there are some who believe that there is no biological aging process as such, and that aging is simply a presentation of chronic disease. This is a difficult dilemma to adjudicate.

To be able to adjudicate this dilemma, we must determine whether a biological pathway or process contributes to aging, and, to do so, we must be able to measure aging itself. The passage of physical time is associated with aging. However, this association is imperfect. Suffice it to say that a comparison of two individuals of the same calendar age may yield very different assessments of their function ability [8], and their time to death may differ as well [9]. These two well-known facts constitute the premises for the construction of the quantity we call biological age. Once we have biological age in hand, it is possible to determine whether the hallmarks of aging, and their derivatives, are true determinants of aging, and we can also resolve the ambiguity inherent in the geroscience hypothesis.

## The frailty index, a quantitative measure of biological age

Quantification of biological age has become a popular topic during the past several years, and there are many ways to do this [10, 11]. However, the search for biomarkers of aging goes back much further [12, 13]. In a related, but separate endeavor, clinicians attempted to stage patients and to extend their findings to aging populations [14]. These attempts led to the first method explicitly touted for this purpose, the frailty or deficit index [15, 16]. The frailty index (FI) is defined as the proportion of deficit items scored out of the total examined. The items examined are physical or cognitive function variables, diseases and disorders, physiological measures, serum analytes, and, more recently, cellular and molecular parameters [15–18]. Items from these categories can be mixed and matched [8, 16, 19–21]. The only requirements are that they change with age and that they signal the status of various body systems. Calendar age is never a deficit item in an FI.

The deficit index increases exponentially with calendar age [18, 22]. Some researchers measure biological age by departure of the individual’s FI from the population mean at any given age [15]. However, this is not necessary. The population average need not enter consideration because the individual’s FI itself is the distinguishing variable [18], simplifying the determination of biological age. Health decreases as FI increases, by definition. Thus, healthy aging can be expressed as the function of 1-FI, because FI is a fraction. It is important to note that there is no necessity of fixing the number or the identity of the items that are included in an FI. Items are selected on the basis of their wide availability in the population under study and by their representation of a broad range of body systems. Some other attributes of the FI can be discussed for the specific example of FI34, where the 34 indicates the total number of items in the index that can be scored [22].

FI34 is a better predictor of survival than calendar age in older adults [22]. In this regard, it is noteworthy that mortality in the world population increases exponentially with age in agreement with the Gompertz equation, but, starting at age 80 and very clearly at age 90, there is a departure from this tendency, such that the observed mortality increases at a decreasing rate [23]. This is precisely the point at which FI34 becomes a better predictor of survival than calendar age [20, 21, 24, 25]. FI34 increases more rapidly with calendar age in the offspring of short-lived individuals than in the offspring of long-lived ones, suggesting that it is heritable [22]. Indeed, twin studies have determined the heritability of FI34 to be about 39% [22]. Hierarchical clustering of the deficits exhibited by the offspring of long-lived as compared to short-lived parents suggests that the patterns of aging differ markedly between long-lived and short-lived individuals, supporting the heritability of the FI [22].

## Bioenergetics and mitochondrial functions in biological aging

FI34 has been used to characterize the biological aging process in more detail. Resting metabolic rate (RMR), which measures the energy consumed for maintenance of basic body functions, decreases with age, as do physical activity energy expenditure and total daily energy expenditure [26]. However, in nonagenarians, RMR increases as FI34 gets larger [26]. This counterintuitive association survives adjustment for relevant covariates. This suggests that it takes more energy to maintain basic body functions in these individuals when they are not healthy [26]. Thus, it is possible to envision biological aging as a loss of integrity and functional coordination that results in increased energy demand [15, 26, 27]. The structure of the FI and the loss of integrated body function with biological age conjure up the view of the aging system as a network. Males and females both display this association of RMR and FI34 [26]. However, in females it is correlated with a loss of lean body mass, while in males it is correlated with a decline in muscle quality. In both sexes, the association of RMR with FI34 is related to mitochondrial function, nevertheless, but in differing ways [26]. Genetic studies have shown that in females the mitochondrial uncoupling protein genes *UCP2* and *UCP3* play a role, suggesting that the energy source (glucose versus glutamate) and the intensity of mitochondrial oxidative phosphorylation are important [28]. In males, the culprits identified are the genes *LASS1* and *XRCC6*, which are involved in mitochondria-dependent cell death[29].

In a genetic linkage study of nuclear families consisting of at least one long-lived parent (≥ 90 years old) and their offspring, a locus in a non-coding region of chromosome 12 was associated with healthy aging using FI34 [30]. This genomic region has three healthy aging-associated sites (HAS), two of which possess the features of enhancers and the third displays the characteristics of a polycomb repressor. One of the enhancer sites had been shown previously to be associated with multiple diseases, some of which are age-related, and was experimentally determined to possess enhancer activity. These results were replicated in a separate population, in which all three HAS were found to contain single-nucleotide polymorphisms (snp) associated with longevity. This study confirms that FI34 is heritable.

The heritability of FI34 was exploited in a twins study for genome-wide analysis of DNA methylation sites and regions associated with healthy aging [31]. The most significant out of the enriched 68 GO terms assigned to genes in the vicinity of the methylation sites were found to be “homophilic cell adhesion via plasma membrane adhesion molecules.” The CpG sites in the 5’-CpG Island of the *PCDHGA3* gene were the most significant. *PCDHGA3* is located in one of three large, closely linked clusters of protocadherin genes on chromosome 5. Combinatorial expression of protocadherin isoforms yields enormous cell diversity in the nervous system. Protocadherins facilitate homophilic cell-cell interactions and mediate intracellular signaling. Methylation in this large gene cluster has been associated with calendar age and age-related phenotypes, and it can modulate gene expression.

## Epigenetic aging clocks compared to frailty index as measures of biological aging

Aging clocks have been devised to measure calendar and biological age [32, 33]. The most extensively studied ones are so-called epigenetic clocks based on the presence or absence of methyl groups on cytosine at CpG sites throughout the genome [34]. Although first developed in human, these DNA methylation clocks have also been employed in mouse studies [35]. They have been applied to demonstrate the rejuvenating effects of various treatments in mice [36]. Over the past decade, DNA methylation clocks have undergone a substantial evolution.

The first widely acknowledged DNA methylation clock was developed by Horvath [37]. It consists of 353 CpG sites selected using elastic net regression to assign calendar age accurately. Strangely, age is also one of the predictor variables in the regression algorithm. This first-generation DNA methylation clock dubbed ‘DNA methylation age,’ along with two derivative second generation clocks called ‘age acceleration difference’ [38] and ‘age acceleration residual,’ [39] claimed to measure biological age. All three of these clocks were compared side by side to FI34, with calendar age as a covariate, to predict survival using Cox proportional hazards regression [40]. Only FI34 met the challenge and significantly predicted survival, while the DNA methylation metrics failed altogether. Interestingly, FI34 was an even better predictor of mortality than calendar age in nonagenarians in these side-by-side comparisons. The gold standard metric of aging is the exponential increase in mortality described by the Gompertz equation. Any measure of biological age must be validated as a predictor of mortality, to provide the ground truth for this attribute.

The performance of DNA methylation clocks as predictors of mortality has improved with the newer generation clocks. These clocks no longer include calendar age among the predictors, and they also include other biomarkers of aging [41, 42], such as serum analytes and various functional measures. These clocks are often trained to predict survival. Several of the clocks have been compared together showing that they partially overlap, but they also account for complementary portions of some of the variation in survival [43].

## Comparison of the Klemera-Doubal equation and frailty index as biological age measures

**Figure 1.**
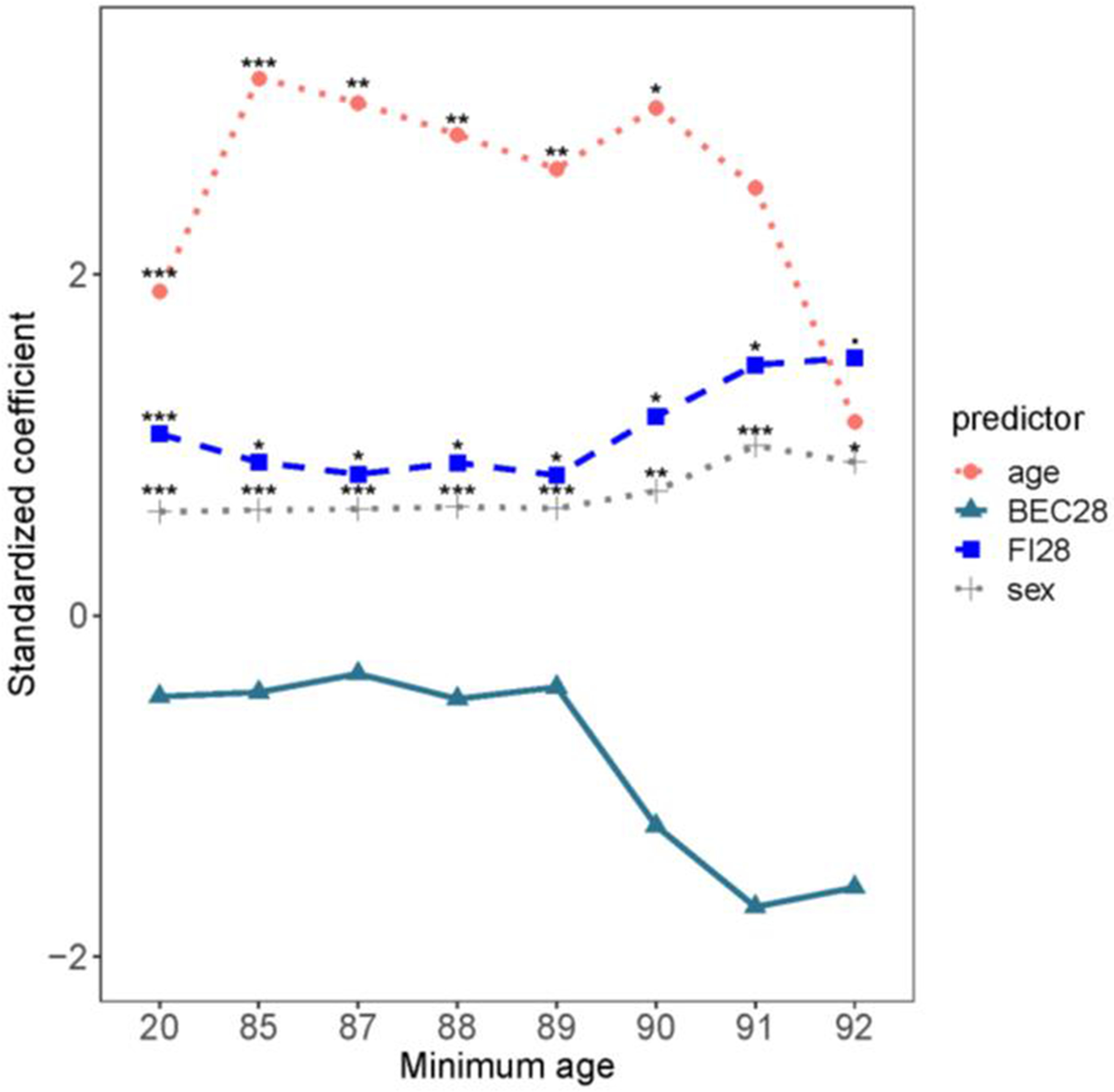
Comparison of FI28 and BEC28 as predictors of mortality. Cox regression analysis was performed with age, sex, FI28 and BEC28 in the same model. The regression coefficients (standardized) are plotted against the age of the participants (*n* = 592) in groups plotted by minimum age of each group, such that in age group 20 all participants are over 20 years-old and for age group 90 all participants are over age 90. The scale on the abscissa is not proportional. ***P ≤ 0.001, **P ≤ 0.01, *P ≤ 0.05. Reproduced with permission from the J. Gerontol. Biol. Sci. Med. Sci. [20].

Another approach to estimation of biological age using biomarkers employs the Klemera-Doubal (KD) equation [44, 45]. This is a very popular approach, often not explicitly applied, because it predicts mortality better than calendar age. However, the KD equation contains two age-derived explanatory variables in the simpler form (BE) and calendar age itself as an additional explanatory variable in the complete model (BEC). The performance of the KD equation in estimating biological age was compared to FI. In this study, the deficit items that were used were selected as an ensemble of the top twenty-eight to predict survival in a combination of three different machine learning algorithms. The selected items entered a simple deficit index, in which the deficits were totaled and the sum divided by 28, yielding FI28 [20]. FI28 is a better predictor of mortality than a BE- or even BEC-type KD-based estimators of biological age that, for explanatory variables utilize the same items from the same population as this FI28 (Figure 1). FI28 outperforms FI34, generated in the customary fashion described earlier, in the same population. Calendar age is a better predictor of mortality in younger adults, in these comparisons. However, its effect size was only larger than that of FI28 up to the lower age threshold of 90 when it became insignificant (Figure 1), reminiscent of FI34 (see above). On the other hand, FI28 was significant throughout the entire age range, and its effect size remained constant (Figure 1).

## DNA methylation index, another form of FI

**Figure 2.**
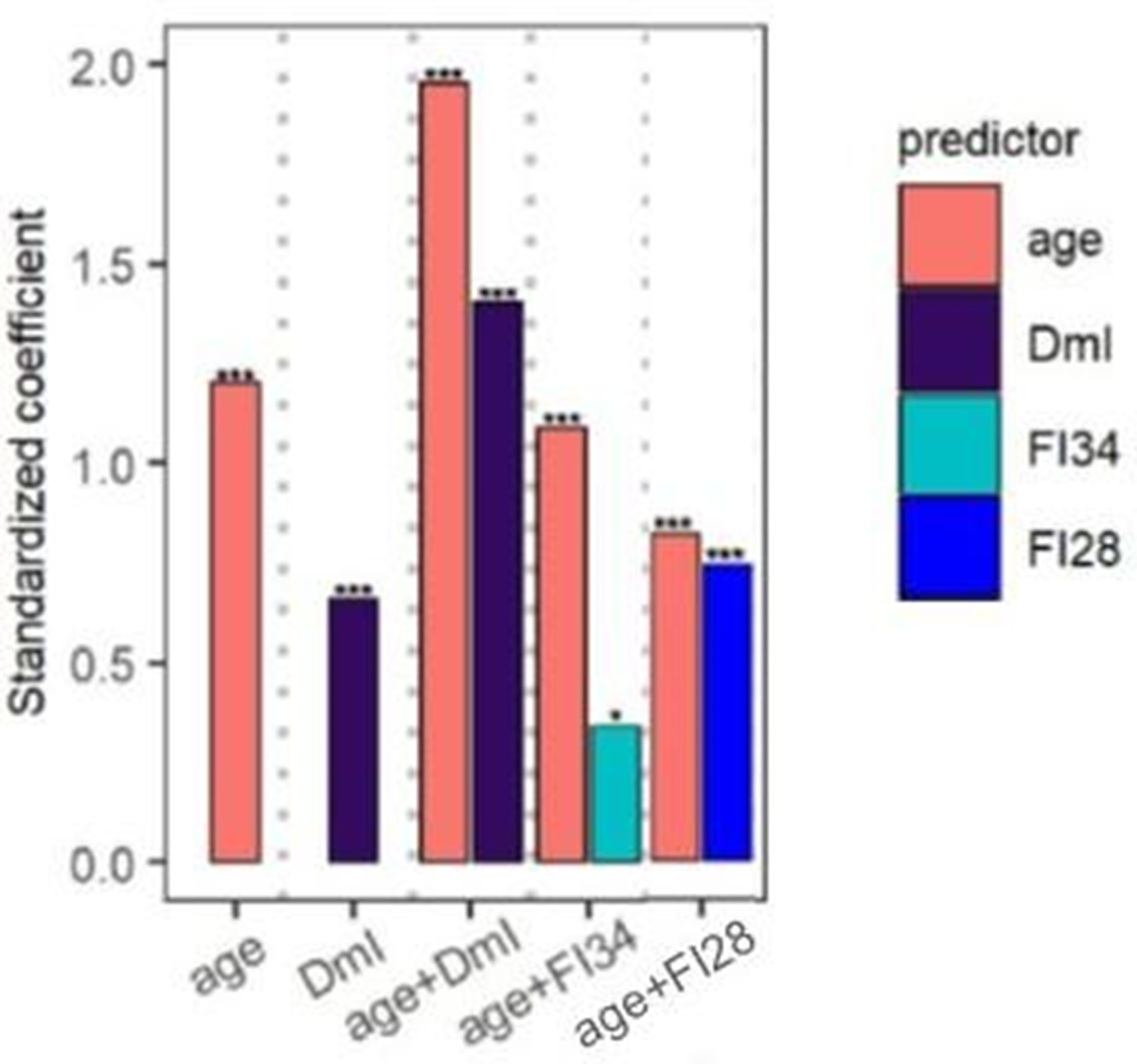
Comparison of FI34 and FI28 with DmI38 as a predictor of mortality. Cox regression analysis was performed with age, DmI, age + DmI, age + FI34, and age + FI28, as indicated, for participants (*n* = 165) in the Louisiana Healthy Aging Study (LHAS). Sex was included in each model. Standardized coefficients are shown. ***P ≤ 0.001, **P ≤ 0.01, *P ≤ 0.05. Adapted with permission from the J. Gerontol. Biol. Sci. Med. Sci. [20].

**Figure 3.**
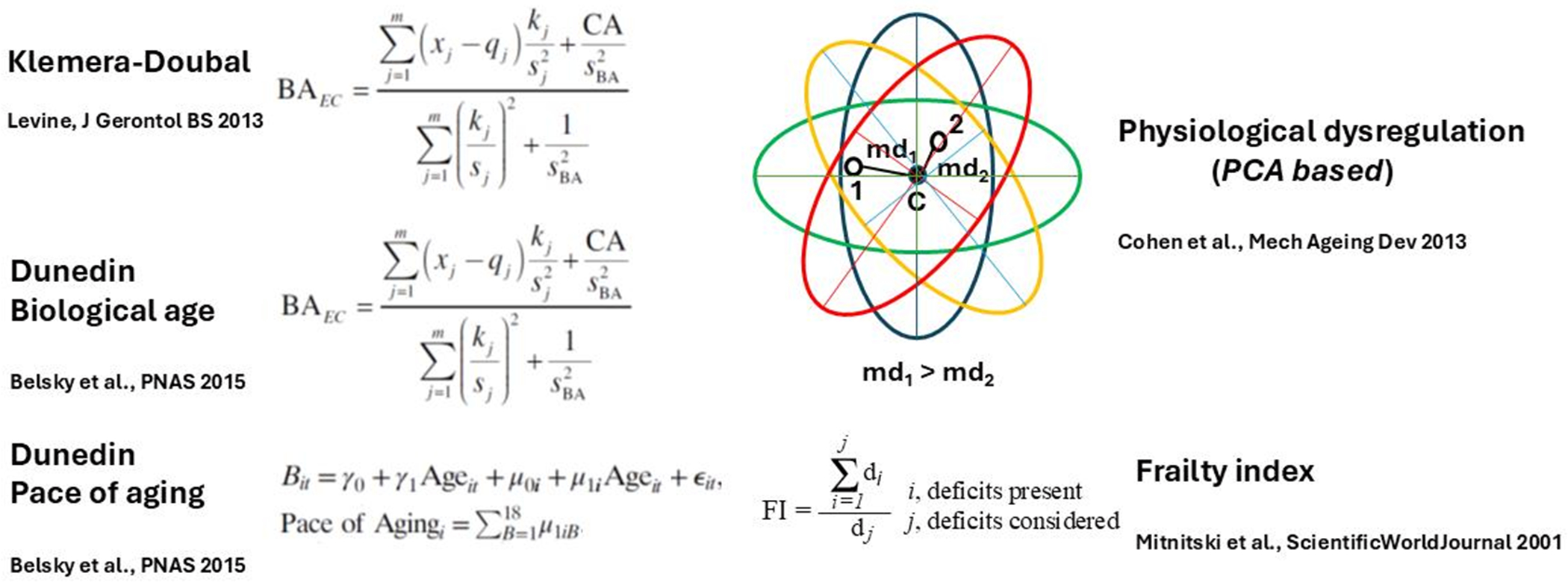
Graphical illustration of the main methods for derivation of biological age (BA). The Klemera-Doubal equation (KD) is shown in its form that contains calendar age (CA) as an explicit variable (B(A)EC). Note that Dunedin Biological age is based on the KD equation. Physiological dysregulation is based on the Mahalanobis distance (md) of a participant from the centroid (d) of the multivariate distributions derived from principal components analysis (PCA) of biomarkers of aging measurements from individuals in a population. References to the literature are shown for each method [16, 45–47].

The procedure for feature (deficit) selection used for generation of FI28 was used to select DNA methylation sites across the genome and compile them into a DNA methylation Index (DmI) composed of 38 CpGs (DmI38) (20). DmI38 was the best predictor of mortality when compared side by side with FI34, FI28, and age (Figure 2). It also outperformed the KD-based measures of biological age [20]. This demonstrates the power of the simplest procedure for measuring biological age by counting deficit/frailty item accumulation. This demonstrates that virtually any characteristic of the aging organism, whether they are biomarkers or health-related items, can be used to derive a metric of biological aging. Several of the most encountered methods for estimating biological age are summarized in Figure 3.

## FI identifies individual variability in aging

**Figure 4.**
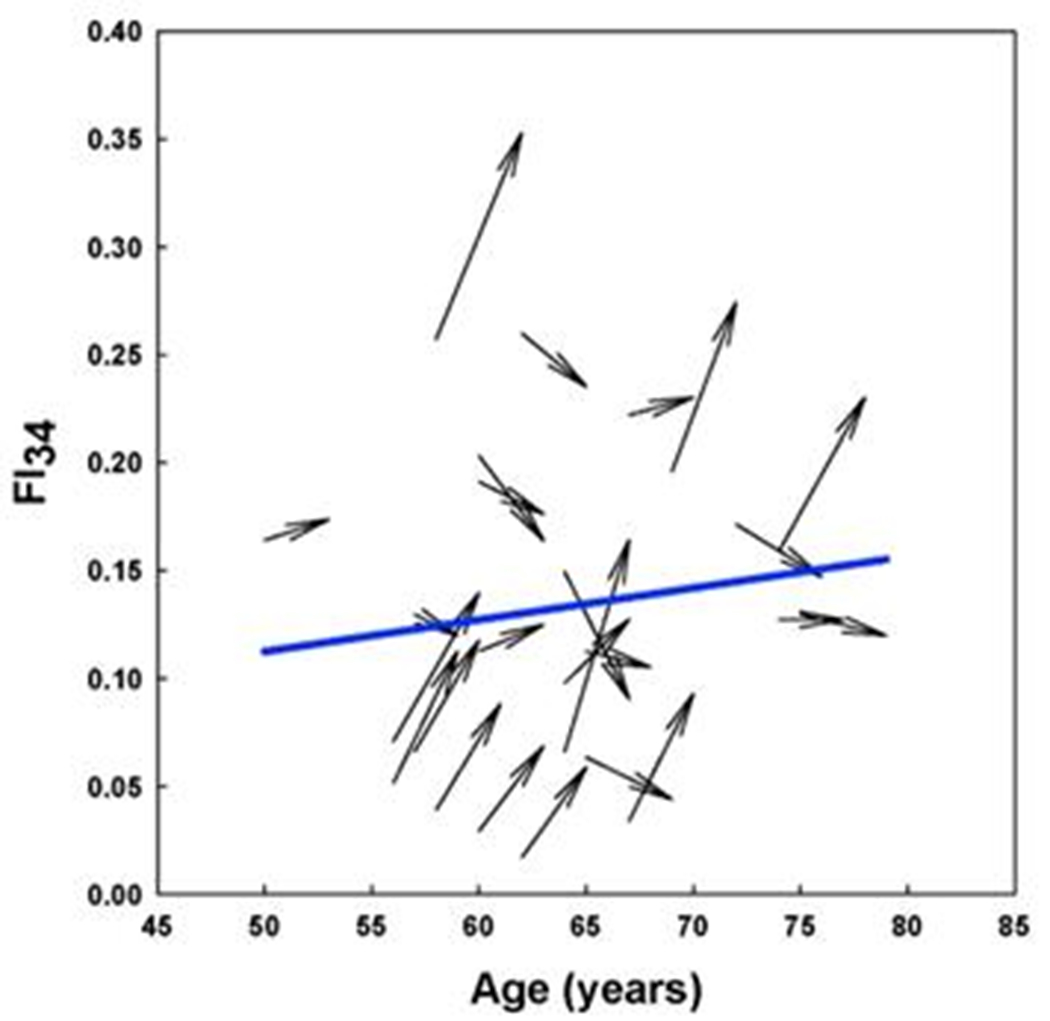
FI28 distinguishes healthy individuals (low FI) from unhealthy individuals (high FI) in an age group. FI28 plotted as a function of calendar age (upper panel). PC biplots for low (left and high (right) FI28 individuals (lower panels) from the stratified age group shown in the upper panel. Colors in upper and lower panels match the indicated population strata. Secondary data analysis from Kim *et al.* [20].

**Figure 5.**
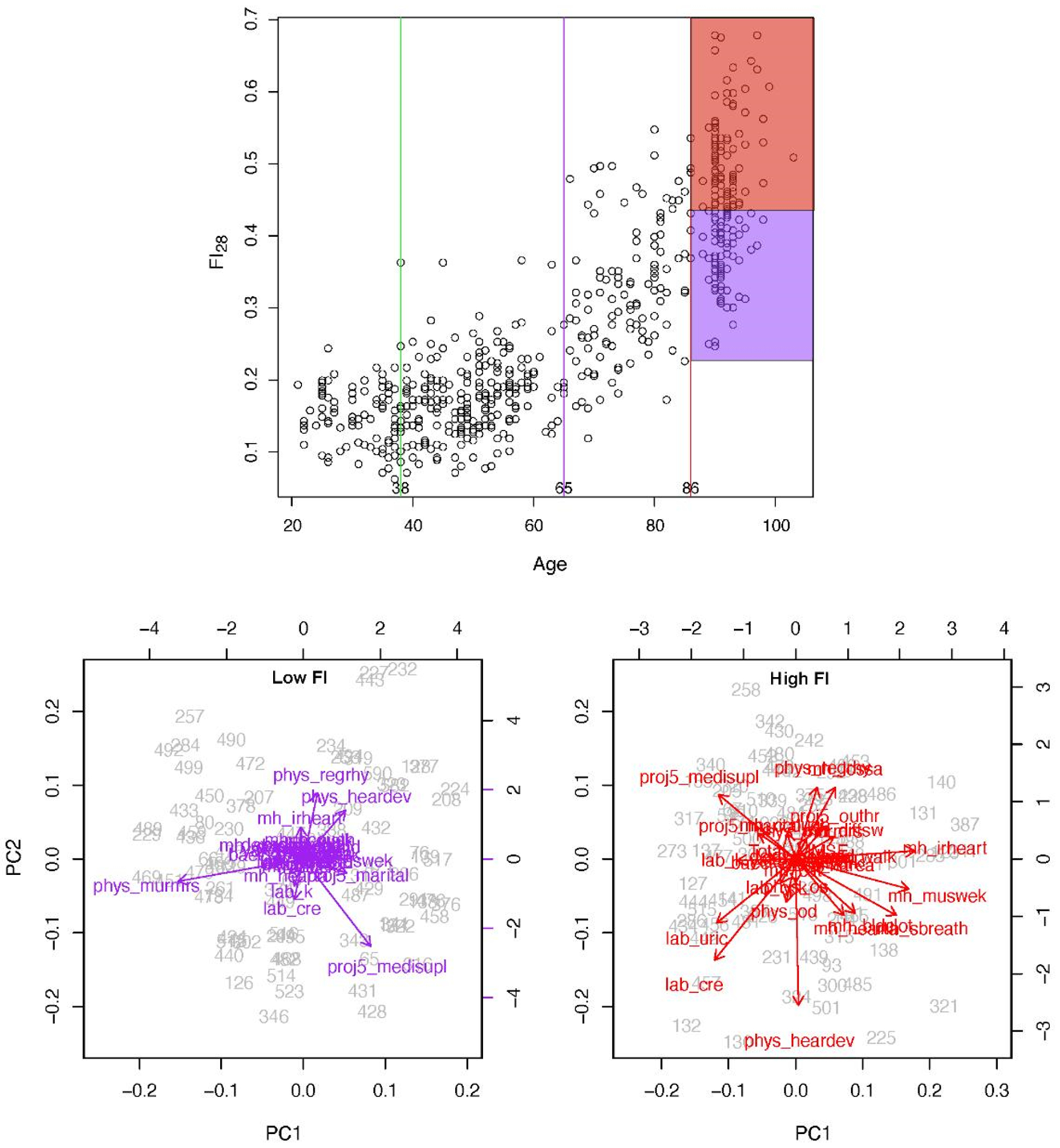
FI28 characterizes the biological age irrespective of calendar age. FI28 plotted by age (upper panel). PC biplots for younger (left) and older (right) by calendar age individuals in lower panels. Colors in upper and lower panels match the indicated population strata. Secondary data analysis from Kim *et al.* [20].

The physiological definition of frailty is like FI in that it relies on tabulation of health-related items [48]. In the Fried measure of frailty, there are only five items: unintentional weight loss, muscle weakness, self-reported exhaustion, slow walking speed, and low physical activity levels [48]. Individuals who score on three or more of these items are considered to be frail, while scoring one or two they are pre-frail, at a heightened risk of becoming frail. The physiological measure of frailty has great clinical value, but the statistical approach of the FI is more adequate as a quantitative measure in biological research [15]. FI allows the classification of individuals according to their biological age and specific characteristics. In Figure 4, FI28 is plotted as a function of calendar age. Individuals in the oldest age group were divided into strata with high and low FI28, with a cut point at the mean. The two strata did not differ by calendar age (*p* = 0.06). Principal Component (PC) biplots show that these two strata differ substantially. On the other hand, when the same cohort was stratified by calendar age into two groups that did not differ in FI28 (*p* ≥ 0.05), their PC biplots were very similar (Figure 5). This comparison suggests that FI, although it is a statistical construct, can discriminate not only the aggregate features of the aging cohort but also the individual characteristics of its members.

**Figure 6.**
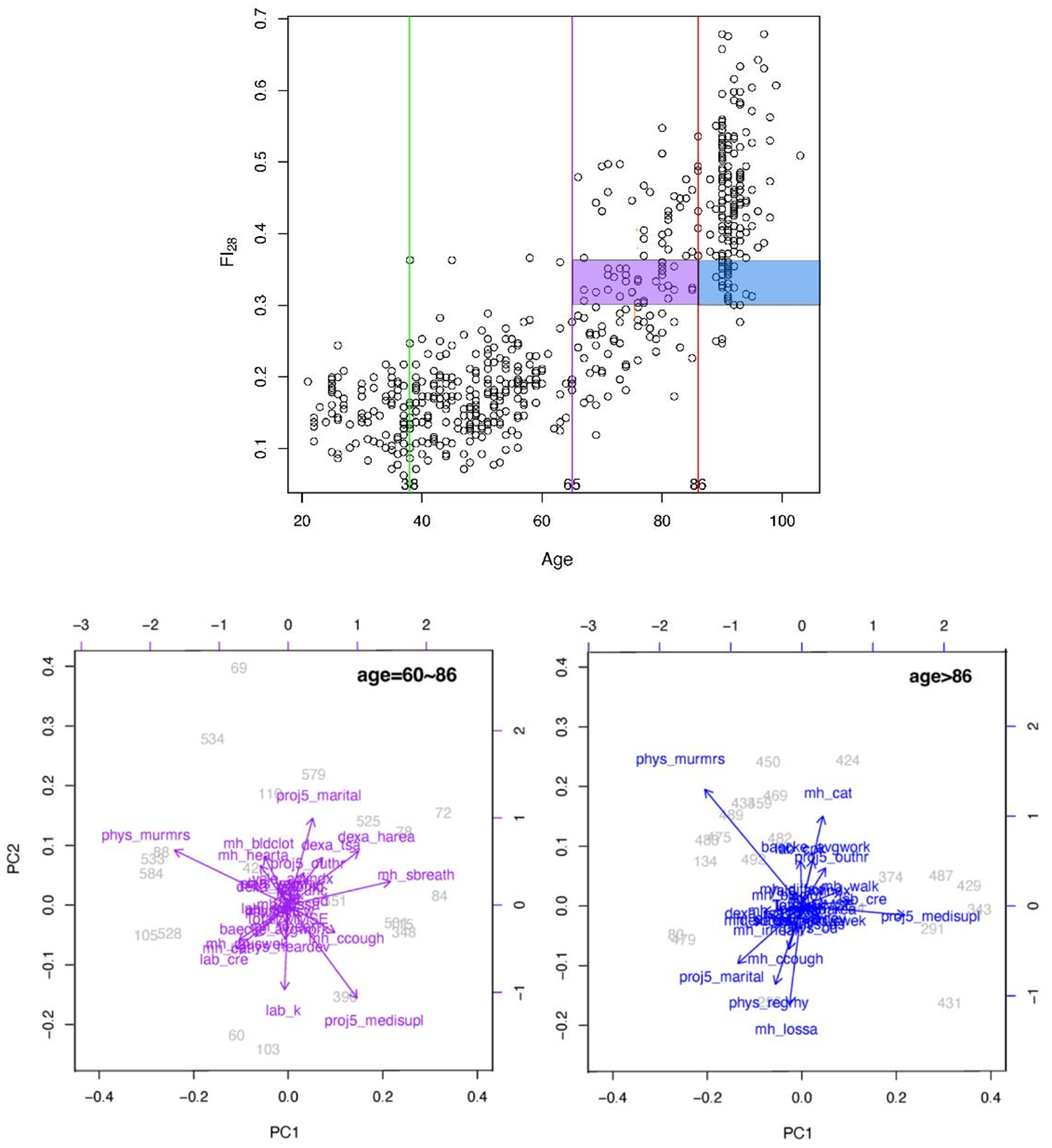
Individual variability in biological age trajectories during calendar aging. Arrows depict the FI34 of individual participants measured at the age shown by the blunt end of the arrow and 3 to 5 years later, as indicated by the pointed end of the arrow. The mean trajectory of FI34 for all the participants is shown by the solid line. Reprinted with permission from Healthy Aging Res. [19].

This power to discriminate between aging individuals is supported by examining individual trajectories of FI34 of an aging cohort (Figure 6). The statistical mean that depicts the biological aging of the cohort decomposes into widely variable individual trajectories of healthy and unhealthy aging [19]. Furthermore, plasticity is evident in the biological aging process, at least over a period of 3 to 5 years. Some individuals inexorably become older and older biologically, while others are rejuvenated to some extent. This raises the possibility of interventions to stall and perhaps even reverse aging.

## The aging system is a network

The hallmarks of aging resemble the deficits that constitute a frailty index in several ways. Both represent functional decline during aging. They both can be gathered from various levels of biological organization: FI deficits can be cellular factors, molecular biomarkers, physical function and/or cognitive function measures; the hallmarks are cellular, molecular, and systemic/organismic processes. Often, they are present concurrently, and they are frequently connected. Indeed, the authors of the hallmarks of aging have stated that the hallmarks are connected. This, together with the observation that FI34 increase in nonagenarians is associated with increased RMR suggesting a decrease in integrated function of the organism, raises the notion that biological aging can be attributed to a deterioration of a network. In fact, a mathematical model of FI as a network that loses critical nodes describes the aging process well [49]. Damage to nodes results in concurrent damage to edges/connections, which is tantamount to loss of integration and coordinated function. This degradation of the network is the result of stochastic damage to its nodes, according to the model. Damage at one point of the network can propagate throughout due to connectivity [50, 51], rendering it difficult to assign initial cause. In this way, the hallmarks of aging appear recurrently across the aging system.

**Figure 7.**
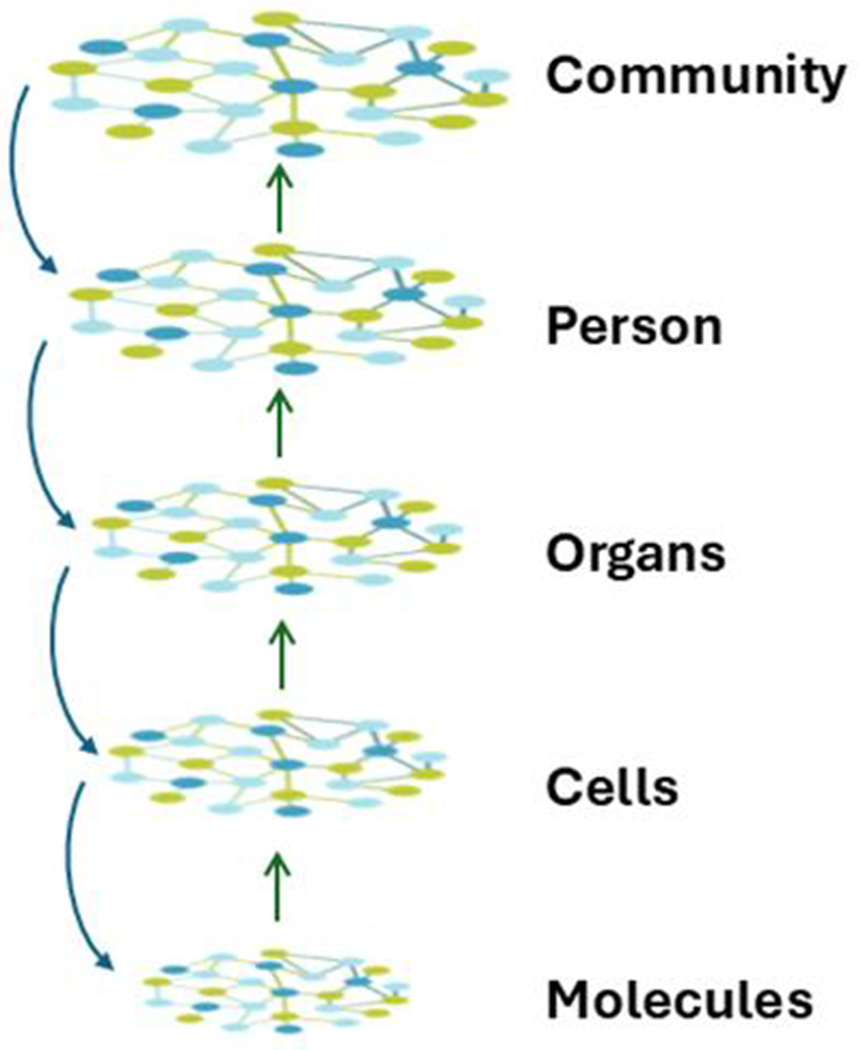
Aging is the systems biology of layered, embedded networks. At the lowest level are networks of interacting molecules. These are embedded within a multitude of subcellular complexes and structures (organelles) forming cells. The many different cell types are organized into various tissues, which in turn are incorporated into organs. Networks, with their myriad connections, constitute the basis for all these increasingly complex levels of organization. In turn, organs form the individual person, who is also a complex interacting supra-network. People exist in communities of individuals who also interact. The space between the individual levels in the network hierarchy can readily be occupied by additional layers, which for clarity are not shown here. The interactions throughout these networks make the system complex, with the level of complexity increasing from the bottom to the top in this figure. This complexity makes the system nonlinear in its dynamics, and results in emergence of new properties that are not simply the sum of the parts. There also is feedback from higher levels of the network hierarchy to the lower levels. The organization of the supra-network is self-similar/fractal and thus may be subject to fractal analysis.

**Figure 8.**
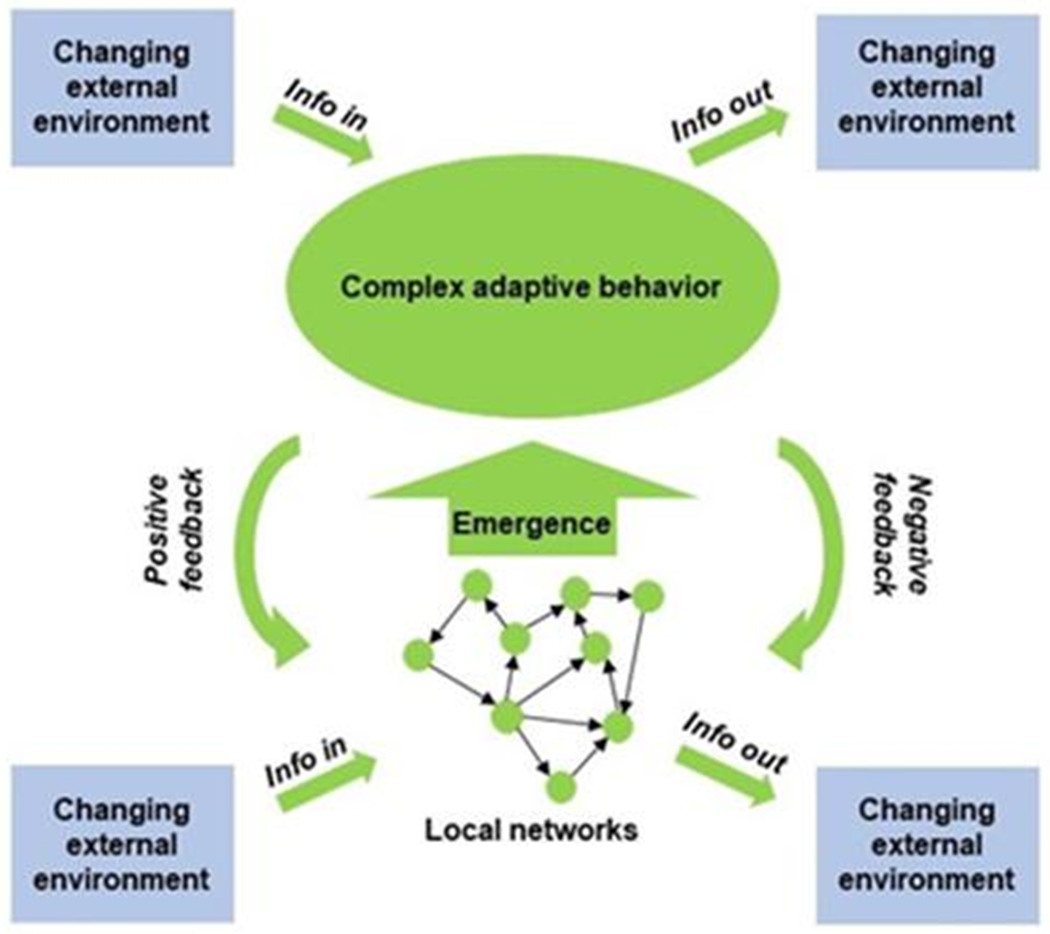
The aging system interacts with the environment at the local and higher network levels. The concepts in this figure are the same as in Figure 7. However, this figure emphasizes the emergence of complex adaptive behaviors, the feedback, both positive and negative, from higher to lower levels of network organization, and the two-way interactions with the environment at various levels in the network hierarchy. Adapted from a file from WikipediA Commons under the Creative Commons CC0 1.0 Universal Public Domain Dedication.

The aging organism is composed of many interconnected networks at all levels of biological organization (Figure 7). Many of the networks at lower levels of biological organization connect with nodes in networks at higher levels. This results in a supra-network, which is simplified in the figure with one arrow going up from each level summarizing many inputs. The state of the network at the higher level affects the operation of the lower levels (arrows downward) that are de facto embedded within it, in a feedback type of mechanism. Because the network is composed not only of nodes but also of edges, it displays nonlinear dynamics [52]. In other words, it constitutes a complex system [53]. Such a system displays emergent properties that cannot be predicted directly by the properties of the individual components [54]. Apparent purpose may be an emergent property of a biological system, as it interacts with its environment in a two-way fashion (Figure 8).

## Functional genomics identifies the networks of an aging system

**Figure 9.**
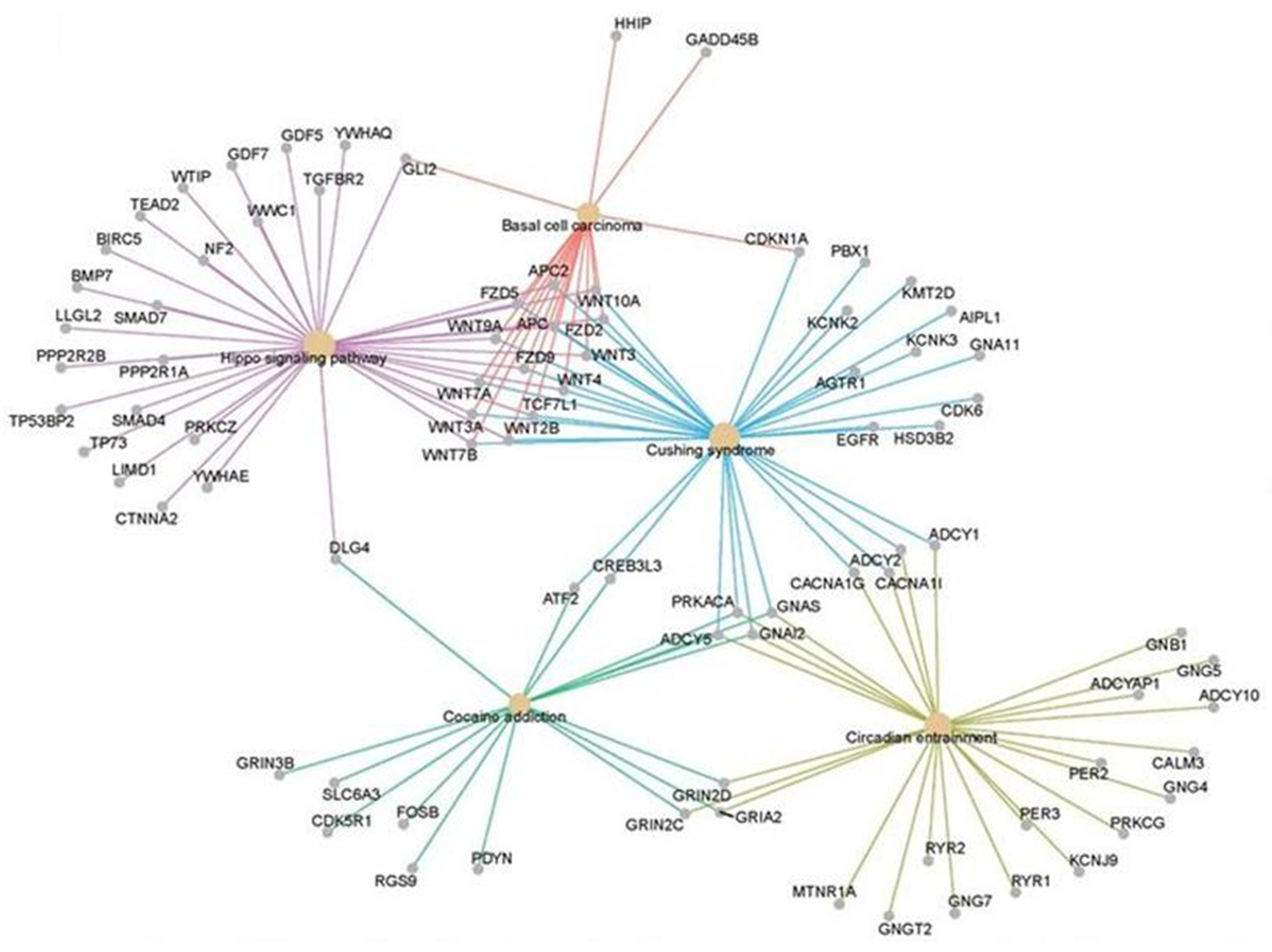
Linked gene regulatory networks associated with biological age. DNA methylation sites (CpG) associated with FI28 were mapped in blood leukocytes from LHAS participants 60 to 103 years of age [40]. The biological functions of the genes linked to these CpG sites were assigned using the Gene Ontology (GO) Consortium database. Functional relationships of the genes in the significant GO categories were inferred from the Kyoto Encyclopedia of Genes and Genomes (KEGG) pathways database [31]. Only the top five gene clusters are shown, all of which have an adjusted *p* < 0.025 (q < 0.023). Secondary data analysis from Kim *et al.* [20].

**Figure 10.**
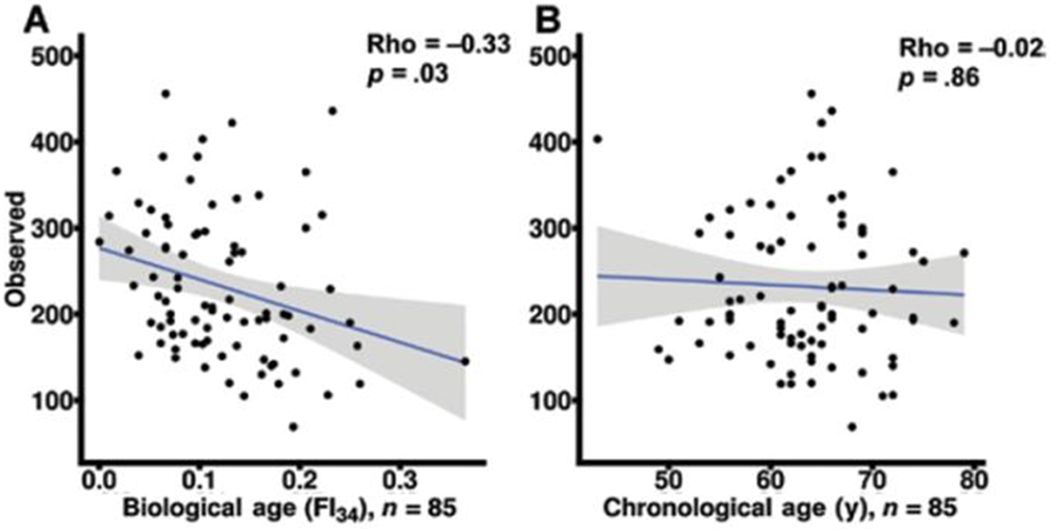
Gut microbiota diversity decreases within individuals with biological age (**A**) but not with calendar age (**B**). Reprinted with permission from the J Gerontol Biol Sci Med Sci [60].

**Figure 11.**
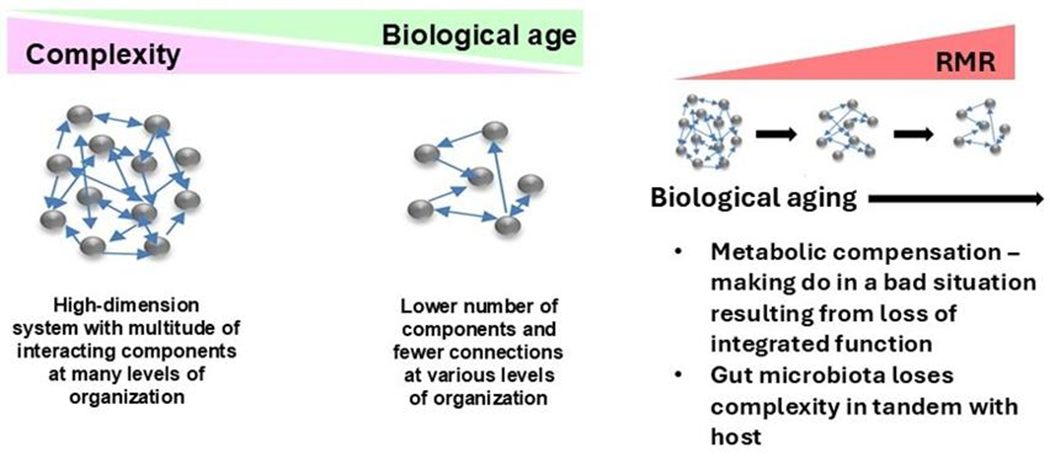
The host and its gut microbiome age in tandem, resulting in loss of complexity which raises energy demands for maintenance of basic body functions. Adapted with permission from Front Genet [25].

Functional genomics can be used to identify networks that define the biological aging process. An unbiased machine learning approach was used to select CpG methylation sites across the genome that predicted FI28 better than calendar age. Functional relationships between genes linked to the selected CpG sites were inferred. (DNA methylation at CpG sites plays a role in gene regulation.) The top five clusters (Figure 9) depict a network that encompasses aspects of brain function, metabolism, and cell proliferation, which highlights the reach of the connections in an aging network. (See also [55]. The epigenome, in which DNA methylation plays a central role, constitutes the interface between the genome and the environment [56, 57]. The gut microbiome plays an essential role at this interface [58, 59]. Interestingly, the gut microbiota shows a decrease in α-diversity (diversity within an individual) with biological age, but not with calendar age (Figure 10). Thus, the microbiome loses complexity just as the host does. They both age in tandem. The result is an increase in the energy needed to maintain basic body functions (Figure 11).

The operation of a system schematically depicted in Figures 7, 8, and 11 is best quantified using stochastic thermodynamics. Such a system is characterized by increasing entropy as it becomes more disorganized [61, 62]. This loss of organization is caused by loss of connections in the network. As entropy increases energy dissipates and becomes less available. This is reflected in the requirement for provision of more energy to maintain basic body functions. Furthermore, the increase in entropy means that there is a higher number of possible microstates, which means greater disorder and the probability that the system will collapse into a certain state. This is the source of the heterogeneity of aging from individual to individual.

## Conclusions

The hallmarks of aging fit snugly within the confines of the aging system modeled as a network. They are the nodes in this network at various levels of organization. They are repeated in different sub-networks that feed into higher level nodes. Any effector that impinges on the supra-network, such as an environmental factor, propagates across the entire network. This often makes it impossible to identify the first cause. It is also possible for local changes in the equilibrium of the network to occur, without the imposition of external factors, to lead to spontaneous and stochastic changes in entropy that propagate. This results in the individual variability of aging, even in genetically identical individuals in a population maintained under the same environmental conditions. A nonlinear dynamic equation modeling the biological aging process, dubbed epigenetic stratification, generates the same net results [53].

The network model of an aging system/organism explains why there are so many changes, diseases, and disorders associated with biological aging. This model also explains the inter- and intra-individual variability in its manifestations. The network model explains the nonlinear dynamics of an aging system and the emergence of unexpected phenotypes due to that complexity. It also accounts for the hallmarks of aging, their recurring appearance at different locations in the system, and the potential for additional, new hallmarks to be defined. Thus, the network model facilitates the analysis of the aging system both as a ‘whole’ as well as the analysis of its subsystems. The model allows us to write deterministic rules that govern the stochastic aging process [6]. The qualitative model described here should be expressed in the language of stochastic thermodynamics to encourage quantitative testing.

## Data Availability

This is a review article. Secondary analysis was conducted on data from Kim *et al.* [20, 31, 40] for Figures 4, 5, and 9.
